# Calcium-Sensing Receptor (CaSR)-Mediated Intracellular Communication in Cardiovascular Diseases

**DOI:** 10.3390/cells11193075

**Published:** 2022-09-30

**Authors:** Hezhen Chu, Zhenqian Qin, Jun Ma, Yimin Xie, Haifeng Shi, Jie Gu, Baiqiang Shi

**Affiliations:** 1 Department of Urology, Yixing Traditional Chinese Medicine Hospital, Yixing 214200, China; 2 Department of Urology, Affiliated Hospital of Jiangsu University-Yixing Hospital, Yixing 214200, China; 3 School of Life Sciences, Jiangsu University, Zhenjiang 212013, China

**Keywords:** calcium-sensing receptor, cardiovascular diseases, nucleus, mitochondria, proteasome, lysosome

## Abstract

The calcium-sensing receptor (CaSR), a G-protein-coupled receptor (GPCR), is a cell-surface-located receptor that can induce highly diffusible messengers (IP3, Ca^2+^, cAMP) in the cytoplasm to activate various cellular responses. Recently, it has also been suggested that the CaSR mediates the intracellular communications between the endoplasmic reticulum (ER), mitochondria, nucleus, protease/proteasome, and autophagy–lysosome, which are involved in related cardiovascular diseases. The complex intracellular signaling of this receptor challenges it as a valuable therapeutic target. It is, therefore, necessary to understand the mechanisms behind the signaling characteristics of this receptor in intracellular communication. This review provides an overview of the recent research progress on the various regulatory mechanisms of the CaSR in related cardiovascular diseases and the heart–kidney interaction; the associated common causes are also discussed.

## 1. Introduction

The calcium-sensing receptor (CaSR) is a recognized member of G-protein-coupled receptors (GPCRs) Class III or Family C and plays multiple roles in animal cells, such as cell proliferation, apoptosis, autophagy, migration, and invasion, which contributes to CaSR signaling as a potential therapeutic target for related physiological disorders, diseases, or even cancer intervention [1,2,3]. However, constant activation of CaSR signaling will lead to Ca^2+^ homeostasis alteration [4].

The CaSR has three typical structural domains of GPCRs: a large extracellular amino (N)-terminal domain, with 612 amino acid residues; a 7 transmembrane domain (TMD) of 250 amino acid residues; and a 216-amino-acid-containing intracellular carboxy (C)-terminal domain [5]. It was revealed that receptor activation engenders intrinsic 7TM asymmetry in the homodimer that promoted G-protein coupling, which was stabilized by calcimimetic positive allosteric modulators but locked by calcilytic negative allosteric modulators [5]. The binding of the negative allosteric modulator (NPS-2143) in 7TMD rearranges the dimeric of two 7TMDs and pushes TM5, TM6, and TM7 away [6]. In addition, Chen et al. found a new Ca^2+^ binding site that stabilized the closure of active Venus flytraps, leading to conformational changes in the 7TMDs to initiate signaling [7]. These structural studies of the CaSR provide insights into the mechanism of CaSR signaling and the potential therapeutic strategies for related diseases. However, these in vitro studies still have limitations, as they cannot identify multiple CaSR-mediated intracellular signals.

Both the extracellular amino (N)-terminal domain and TMD participate in Ca^2+^ sensing. The cytoplasmic C-terminal domain forms dimers and couples with the G-protein subtypes, including Gα_q/11_, Gα_i/o_, Gα_12/13_, and Gα_s_, to induce various signaling pathways. For example, Gα_q/11_ activates the phospholipase C (PLC)/inositol 1, 4, 5-trisphosphate (IP3)/IP3 receptor/endoplasmic reticulum (ER)-released Ca^2+^ pathway, the PLC/diacylglycerol (DAG)/protein kinase C (PKC)/MAPK pathways, as well as the phospholipase A_2_ (PLA_2_)–arachidonic acid (AA) pathway [8,9]. CaSR-activated Gα_i/o_ inhibits the adenylate cyclase (AC)/cyclic AMP (cAMP) pathway [8]. Gα_12/13_ induces Rho/PLD-mediated cytoskeletal remodeling [10,11], and Gα_s_ elevates cAMP levels [12]. However, as these studies investigated CaSR signaling in limited cell lines, they only focused on parts of the whole picture. 

These CaSR signaling pathways are dependent on the expression tissues of the CaSR. The CaSR is expressed in both calcitropic tissues such as the parathyroid glands, the kidneys, and the bone, and non-calcitropic tissues such as the lung, neurons, and heart [13]. The CaSR plays an important role in cardiovascular physiology, and the dysregulation of CaSR signaling participates in cardiovascular diseases [14]. In the vascular endothelial cell, extracellular Ca^2+^ activates the CaSR to increase the cytosolic Ca^2+^ level through intracellular Ca^2+^ release from the endoplasmic/sarcoplasmic reticulum (ER/SR) and extracellular Ca^2+^ entry [15,16]. However, whether the CaSR-induced increase in the cytosolic Ca^2+^ level caused the mitochondrial Ca^2+^ overload in vascular endothelial cells was not identified. The Ca^2+^ overload in the mitochondrial of vascular endothelial cells is associated with the loss of mitochondrial membrane potential, impaired mitochondrial respiration, and the excessive generation of mitochondrial reactive oxygen species (ROS) [17,18]. As one of the functions of the CaSR is Ca^2+^ redistribution and homeostasis, its role in vascular endothelial cells should be further investigated. A comparison between the spontaneous calcium transients and calcium handling proteins from human-induced pluripotent-stem-cell (hiPSC)-derived cardiomyocytes and those from human embryonic stem cells (hESCs) indicated that calcium homeostasis is a key mechanism underlying the calcium handling properties of hiPSC-derived cardiomyocytes [19]. However, this has not been further investigated in terms of the regulators of calcium homeostasis. In mouse ESCs, the CaSR is functionally expressed, and the activation of the CaSR is involved in the differentiation of mESCs into cardiomyocytes, while the differentiation rate was abolished by the inhibition of the CaSR, phospholipase C, the IP3 receptor, and Ca^2+^-ATPase, or by the depletion of the SR Ca^2+^ store [20]. This should be reinforced by further confirmation of in vivo results. In acute myocardial infarction (AMI) rats, CaSR expression was increased over time, and the inhibition of the CaSR by Calhex231 enhanced the efficacy of mESC transplantation for the treatment of AMI by inhibiting apoptosis and oxidative stress [21]. The effects of the CaSR inhibitor Calhex231 on the differentiation of mESCs have not been investigated, and the complications of the xenotransplantation of mouse cells into rats also need to be considered.

However, due to the highly specific cell-type specificity of CaSR regulation and signaling, the pharmacotherapy of cardiovascular diseases that directly target the CaSR may have adverse effects on other tissues, such as kidney injury. The CaSR has a pivotal role in maintaining Ca^2+^ homeostasis in the kidneys, and the activation of the CaSR might protect against kidney injury or chronic kidney disease (CKD) [22,23]. In mouse renal tubular cells, the activation of the CaSR by NPS R-467 could restore the intracellular Ca^2+^ level by switching the PLC pathway [22]. However, the mechanisms of the activation of the CaSR in regulating intracellular Ca^2+^ redistribution and homeostasis have not been investigated. There is considerable interest to use stem and progenitor cells, such as mesenchymal stem cells (MSCs), ESCs, iPSCs, and kidney-derived stem/progenitor cells (KSPCs) to recapitulate kidney development and restore renal function [24]. However, more preclinical studies are required because the molecular, morphological, and functional characterizations of the “kidney cells” generated from these cells have not been exhaustive. The long-term safety of renal stem cells and potential negative effects such as tumor formation also need consideration. In metanephric mesenchymal cells, the spontaneous calcium activity is dependent on the release of Ca^2+^ from the ER store and acts as a stochastic factor for the self-organizing process that controls branching morphogenesis and determines the ultimate number of nephrons in the kidney [25]. Since the CaSR has a central role in inducing Ca^2+^ release from the ER store, the regulation of the CaSR may play an important role in the branching morphogenesis of metanephric mesenchymal cells. In addition, the CaSR can protect podocytes from stress-related death and also mediates dysfunctional integrin signaling and the potassium channel function in podocyte-like cells derived from the induced pluripotent stem cells in Alport syndrome patients [26]. The limited sample size and the in vitro studies may not truly show the functional properties of podocytes in situ. Due to the multiple roles of the CaSR in kidney diseases, it is necessary to understand the potential of targeting the CaSR as a therapeutic approach in cardiovascular disease patients with cardiorenal syndrome.

The CaSR-regulated cytosolic and organellar Ca^2+^ levels activate various pathways, leading to two different endpoints: the sublethal (possibly reversible) or lethal (cell death) changes responsible for subsequent reactions to injury, such as remodeling (i.e., hypertrophy) and repair (i.e., diabetic cardiomyopathy and fibrosis). The disruption of Ca^2+^ homeostasis contributes to various types of cell death: (a) abnormal ROS production; (b) abnormal cytoskeletal contraction leading to catastrophic cell architecture disorganization; (c) the improper activation of cellular proteases (proteasomes, caspases, calpains, matrix metallopeptidases, etc.), leading to the rapid irreversible degradation of crucial substrates; (d) pore formation in the plasma membrane leading to pyroptosis and necroptosis [27,28,29]. The regulation of the CaSR on these types of cell death has been investigated in cardiovascular and kidney cells, as well as some other cell types [30,31,32]. Thereby, the clarification of the cellular signaling of the CaSR may help to treat cardiovascular diseases in the future. This review focuses on the CaSR-mediated intracellular communication in the non-calcitropic cells of the cardiovascular system and calcitropic kidney cells and the diverse regulation of CaSR signaling in protecting or promoting cardiovascular diseases. We present a general overview of CaSR signaling between the mitochondria, ER, nucleus, lysosome, and proteasomes in related cardiovascular diseases. In addition, the CaSR signaling pathways involved in various common causes of cardiovascular diseases are discussed based on the available data.

## 2. CaSR Signaling in Cardiovascular Diseases

The CaSR is expressed in different types of cardiovascular cells, such as vascular smooth muscle cells (VSMCs), endothelial cells, and ventricular cardiomyocytes, in which it plays important roles in the regulation of proliferation, blood pressure, and blood vessel tone [33,34]. To identify the role of the CaSR in related cardiovascular diseases, some in vivo and in vitro studies have been performed. In the following sections, we discuss the CaSR-mediated intracellular communication in several classical cardiovascular diseases, including ischemia/reperfusion damage, myocardial hypertrophy, diabetic cardiomyopathy, and cardiorenal syndrome, which is associated with the heart–kidney interaction.

### 2.1. Ischemia/Reperfusion Damage

Ischemia/reperfusion increases the intracellular and mitochondrial Ca^2+^ levels by reducing active Ca^2+^ reuptake into the ER and ATPase-dependent Ca^2+^ efflux across the plasma membrane [35]. The CaSR induces Ca^2+^ release from the ER and its transfer into the mitochondria, leading to mitochondrial depolarization, which is mediated by the mitochondria-associated ER membranes (MAMs), and it is a potential treatment target in cardiac ischemia–reperfusion injury and heart failure [36,37]. The increasing free radicals released from the mitochondria further aggravate ER stress. This indicates that protein kinase C (PKC) plays a central role in the CaSR-mediated communication between the mitochondria and the ER. The CaSR can be phosphorylated by PKC, which regulates cell growth, homeostasis, and programmed death. PKC phosphorylates the CaSR, which will cause the desensitization of the CaSR, and as a feedback mechanism, the activation of the CaSR also increases the phosphorylation of PKC. PKC-δ translocation from the membrane to the cytosol was found in the ischemic heart. Additionally, in the isolated mitochondria from hearts that had undergone ischemia/reperfusion (I/R) combined with the CaSR agonist GdCl_3_, a significant increase was observed in phosphorylated PKC-δ translocation to the mitochondria, as well as an increased release of cytochrome c from the mitochondria with an obvious decrease in the mitochondrial potential [38,39]. As the CaSR is sensitive to the environmental Ca^2+^ levels, its function as a buffer or its operation during the isolation process of the heart may also modulate CaSR-mediated intracellular Ca^2+^ signaling and homeostasis.

PKC-ε interacts with the CaSR in the ER–mitochondria crosstalk, which might be a potential target to prevent I/R-induced cardiovascular injury, and the underlying molecular mechanism can be further identified at a cellular level. PKC-α has been shown to be associated with the crosstalk of mitochondrial and ER stress in human hormone-refractory prostate cancer cells [40], and PKC-ε interacts with the CaSR to protect apoptosis via inhibiting the mitochondrial Ca^2+^ overload and the disruption of the mitochondrial function induced by the ER in ischemic post-conditioning cardiomyocytes [41]. It has been reported that I/R increased the expression of the CaSR and cardiomyocyte apoptosis, and the activation of the CaSR by GdCl_3_ induced PKC-δ translocation into the ER to induce ER stress-associated apoptotic pathways [42,43]. However, there is still no direct evidence to prove that PKC-δ interacts with the CaSR to regulate the ER–mitochondria crosstalk in the ischemia/reperfusion heart and cardiomyocytes.

In addition, PKC-ε translocates from the cytosol to the nucleus in the ischemic heart during cardiogenesis [39,44], while the function of the nuclear translocation of PKC-ε in cardiovascular diseases has not been identified. It was also observed that PKC-α and PKC-ε were proteolyzed by calpains in the ischemic heart and myocardium [45,46]. Calpain is an apoptotic protein in the mitochondria, and the nuclear translocation of calpain-2 promotes cardiomyocyte apoptosis and remodeling [47,48,49]. Moreover, PKC-α signaling by the calpain-generated free catalytic domains induces HDAC5 nuclear export and regulates cardiac transcription [50]. In these studies on tail-suspended rats, the results suggested that high-dose isoproterenol activated the CaSR by increasing the phosphorylation of the phospholamban of the nuclear envelope and increasing the intranuclear Ca^2+^ transients. However, the mechanism of the CaSR in the regulation of calpain translocation (from the mitochondria to the nucleus) involved in the mitochondria–nucleus crosstalk in cardiomyocytes has not been investigated.

**Figure 1:**
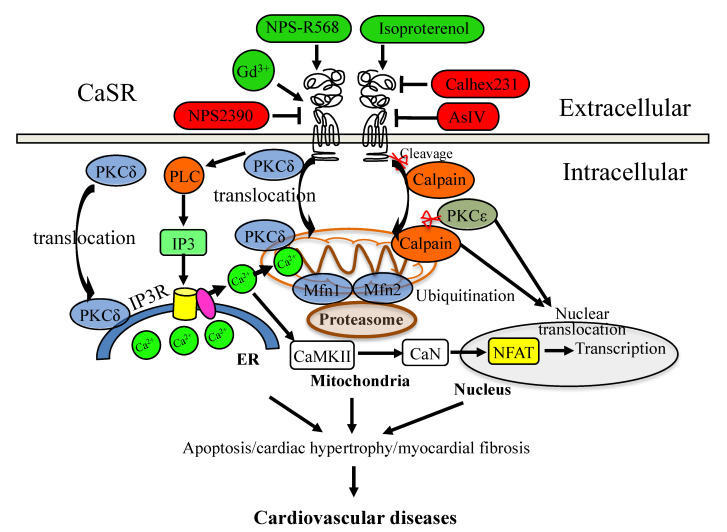
CaSR signaling mediates cardiovascular diseases. The activation regulator is in red color, and the negative modulator is in green color.

The results of in vitro and in vivo studies in spinal cord neurons showed that the expressions of the CaSR and calpain increased during the spinal cord ischemia–reperfusion injury (SCIRI), and the expressions of calpains were enhanced or decreased by the CaSR agonist GdCl_3_ and antagonist NPS-2390, respectively [51]. The CaSR inhibitor NPS-2390 protects cardiomyocyte apoptosis by decreasing the CaSR-regulated calpain expression [52]. These studies indicate that the CaSR interacts with PKC and calpain proteins, which can translocate from the membrane to the mitochondria and/or to the nucleus in I/R hearts and cardiomyocytes; however, the mechanism of the CaSR–PKC/calpain pathway in the ER–mitochondria and mitochondria–nucleus communication, and its roles in cardiovascular diseases still need further studies (Figure 1).

### 2.2. Cardiac Hypertrophy

Hypertension is one of the cardiovascular risk factors for hypertrophy and the development of heart failure. Chronic hypertension causes left ventricular hypertrophy, which ultimately results in heart failure. Previous studies revealed that the CaSR could be a potential therapeutic target for hypertension. In spontaneously hypertensive rats (SHRs), calcimimetic R568 administration reduced blood pressure and myocardial remodeling and reversed the low expression of the CaSR [46]. As a negative allosteric modulator of the CaSR, Calhex231 decreased the heart-to-body weight ratio and the protein levels of the CaSR and attenuated myocardial apoptosis during hypertension [53]. In these two studies, they applied only one modulator of the CaSR as treatment, but it is interesting that both the activation and inhibition of the CaSR could protect the myocardia against hypertension.

Furthermore, previous studies suggested that the regulation of the CaSR could target the mitochondria and ER during cardiac hypertrophy. The inhibition of the CaSR by Calhex231 maintained the mitochondrial dynamic and function through regulating fission and fusion proteins in SHRs [53]. In the cardiac hypertrophy and heart failure model of Wistar rats, the subcutaneous injection of isoproterenol activated the CaSR, increased Ca^2+^ concentration in the mitochondria, decreased the mitochondrial membrane potential, and induced the release of cytochrome c from the mitochondria during ER stress and apoptosis [54]. The CaSR might mediate the Ca^2+^ transfer from the ER to mitochondria and regulate the mitochondrial dynamics, which contribute to cardiac hypertrophy and hypertension.

The CaSR-induced Ca^2+^ release from the ER could activate some Ca^2+^ binding proteins, such as calcineurin (CaN) and the Ca^2+^/calmodulin-dependent protein kinase type 2 (CaMK-II), which further promote the canonical nuclear factor of activated T cells (NFATs) to translocate into the nucleus to regulate the expressions of target genes. In the isolated neonatal rat ventricular myocytes, the CaSR specifically modulated nuclear Ca^2+^ signaling through the IP3R pathway, and the CaSR agonist GdCl_3_ induced cardiomyocyte hypertrophy through the CaN–NFAT pathway [55]. In the same cells, astragaloside IV (AsIV) attenuated cardiac hypertrophy and apoptosis and protected against cardiac injury by reducing the expression of the CaSR, reducing Ca^2+^ release from the ER through the PLC–IP3R pathway, and promoting Ca^2+^ reuptake into the ER through the sarcoplasmic reticulum Ca^2+^-ATPase2a (SERCA2a) pathway, suppressing the activation of the CaMK-II and CaN pathways and inhibiting NFAT-3 nuclear translocation [56]. The results in SHRs suggested that sodium ferulate protected against hypertension-induced cardiac hypertrophy by inhibiting the CaSR-mediated CaN-NFAT-3 signaling pathway [57]. Although these studies had not identified the downstream effectors of the CaSR-mediated CaN-NFAT-3 signaling pathway, they confirmed that the CaSR could regulate the ER–nucleus communication in hypertension-induced cardiac hypertrophy (Figure 1). It is worth noting that the CaSR may also mediate the crosstalk between the mitochondria and nucleus, as the nuclear translocation of mitochondria-related protein PKC-α has been found to induce hypertrophy in neonatal rat ventricular myocytes [58], and the calpain-2 mediated apoptosis of hypertrophied cardiomyocytes has also been observed in transverse aortic constriction rats [59].

### 2.3. Diabetic Cardiomyopathy

Diabetic cardiomyopathy (DCM) is a severe complication of type 1 and type 2 diabetic (T1D/T2D) patients characterized by the human pathophysiological conditions of dyslipidemia, hypertension, and coronary artery disease and results in heart failure. Myocardial fibrosis is a main pathological feature of DCM. High glucose is a common treatment in the studies of diabetic models. In T1D rats and primary neonatal rat cardiac fibroblasts, it was observed that high glucose induced myocardial fibrosis [60]. In cardiac fibroblasts, high glucose also caused excessive cardiac fibroblast proliferation and extensive collagen deposition [61]. Interestingly, the effects of high glucose on the expression of the CaSR depended on cell types. In T1D rats and the primary cultured neonatal rat cardiomyocytes, the CaSR was suppressed by high glucose [62,63], which is contrary to the effects of high glucose in cardiac fibroblasts [61]. However, the mechanism of the opposite effects of high glucose on the expression of the CaSR in cardiomyocytes and cardiac fibroblasts is still unclear, which may promote cell differentiation and contribute to myocardial fibrosis in DCM.

A high-fat diet induces the proteolytic cleavage of the CaSR by calpain, which contributes to the macrovascular late complications associated with diabetes [64]. The ubiquitin–proteasome pathway is another main proteolytic process, which can also be regulated by the CaSR. Previous studies suggested that the inhibition of the CaSR by Calhex231 could alleviate high-glucose-induced myocardial fibrosis via inhibiting the ubiquitin–proteasome pathway [60,61]. Furthermore, the CaSR also regulated mitochondrial function through the ubiquitin–proteasome pathway. The activation of the CaSR protected against a high-glucose-induced decrease in the mitochondrial fusion-related (Mfn1, Mfn2) protein levels via blocking the gp78-ubiquitin-proteasome pathway [65]. By targeting the gp78-ubiquitin-proteasome system, the supplement of spermine downregulated the expression of the CaSR following the decreased ubiquitination levels of mitochondrial fusion-related proteins (Mfn1, Mfn2), which in turn protected cardiomyocytes from high-glucose-induced energy disturbance [66]. These studies suggested that the regulation of CaSR-mediated protease/proteasome signaling with the mitochondria might be a potential treatment for DCM.

## 3. CaSR-Mediated Signaling in Heart-Kidney Interaction

Treatment for cardiac disease patients with kidney injury is particularly challenging, and the damage and dysfunction of one system are often associated with the dysfunction of the other through organ crosstalk. This observation in the clinical diagnosis is defined as a cardiorenal syndrome (CRS), which represents the crosstalk between heart failure and kidney injury and vice versa, as well as alterations in the inflammatory molecules of its clinical phenotypes [67]. For example, interleukin-6 (IL-6) was found to be associated with left-ventricular hypertrophy and systolic dysfunction in patients with CKD, and a high level of IL-6 in CKD contributed to chronic inflammation and subsequently left-ventricular dysfunction [68,69]. The tumor necrosis factor (TNF) and IL-10 contributed to cardiac remodeling and cardiovascular phenotype in the arteries of children with CKD [70].

Acute and chronic heart failure triggers acute kidney injury (AKI) and chronic kidney disease (CKD), which are defined, respectively, as type 1 and type 2 CRS. Types 3 and 4 CRS are the primary insults of renal injury with secondary cardiac dysfunction, and type 5 CRS reflects a systemic condition (e.g., sepsis) simultaneously causing both cardiac and renal dysfunction [71,72]. Recently, the CaSR was considered to be a novel target for the treatment of sepsis-induced CRS (type 5 CRS), as the CaSR mediates sepsis-induced oxidative stress, inflammation, apoptosis, and cardiorenal dysfunction [73]. In addition, the sepsis-increased CaSR expression in T lymphocytes (circulating or resident in heart or kidney tissues) promoted the release of TNF-α and IL-4 cytokines and induced tissue apoptosis and injury [74]. The CaSR mediates a series of pathways involved in CRS, which are described in the following sections.

### 3.1. Inflammatory-Reparative Response

Previous studies have demonstrated that the CaSR mediated the inflammation that influences the cardiovascular system, such as atherosclerosis, vascular calcification, myocardial infarction, hypertension, and obesity [75]. After acute myocardial infarction (AMI), the cardiac system undergoes a repair process, which is mediated by cytokines and inflammatory cells that infiltrate the infarcted sites and require an optimally orchestrated inflammatory-reparative response (IRR) [76]. IRR is indicated as a pathway constantly associated with the CaSR because of the following factors:

(a) Ca^2+^ or other CaSR agonists directly induce the activation of the inflammasome. The activation of the CaSR induced Ca^2+^ release from the ER stores through PLC-IP3 signaling and also reduced the intracellular cAMP, and both of them promoted the assembly of inflammasome components, which was reduced by the knockdown of the CaSR [77]. In addition, the AsIV-inhibited CaSR could suppress the high-glucose-induced NLRP3 inflammasome in human umbilical vein endothelial cells (HUVECs) [78]. The Calhex231-inhibited CaSR decreased the NLRP3 inflammasome activation and IL-1β release in neutrophils from patients with AMI [79]. The CaSR also mediated the inflammatory response in the kidney. The elevated extracellular calcium levels from necrotic cells recruited the monocytes/macrophages that express the CaSR to the kidney injury sites, and Ca^2+^ activated the CaSR to stimulate the assembly of inflammasomes resulting in the maturation of proinflammatory cytokine IL-1β by caspase-1 [80]. The activation of the CaSR by R-568 increased the renal injury in streptozotocin-induced diabetic rats associated with the promotion of the inflammatory response by enhancing the proinflammatory cytokine interleukin-6 (IL-6) levels and reducing the anti-inflammatory cytokine IL-10 levels [81], while an excessive inflammatory response may activate the proapoptosis and remodeling of cardiomyocytes, induce matrix degradation, and impair collagen deposition, which will influence the healing process.

(b) Another reason is the release of damage-associated molecular patterns (DAMPs) by cells under Ca^2+^-mediated stress or death. Clinical injury caused a systemic inflammatory response syndrome by sepsis, and cellular injury could release endogenous DAMPs into the circulation, which activated Ca^2+^ signaling and the MAPK pathway of polymorphonuclear neutrophils, thus leading to migration and degranulation and eliciting neutrophil-mediated organ injury [82]. As a DAMP, the high-mobility group box 1 (HMGB1) is involved in a large variety of different processes, such as inflammation, migration, invasion, proliferation, differentiation, and tissue regeneration, and it also serves as a regulator of inflammatory-reparative responses following MI, by inducing endothelial cells and modulating cardiac fibroblast activity [76,83].

(c) The activation of CaMK-IIδ pathways in response to pressure overload triggered inflammatory NLRP3 and IL-1β gene expressions and increased the caspase-1 activity and the IL-18 cleavage of the inflammasome in cardiomyocytes [84,85]. Additionally, targeting CaMK-IIδ might prevent cardiac ischemia/reperfusion injury by inhibiting myocardial inflammation [86,87]. Although the regulation of the CaSR on CaMK-II in cardiac hypertrophy and apoptosis has been investigated, the role of this special isotype, CaMK-IIδ, in CaSR-regulated cardiac inflammation still needs to be identified.

(d) The CaSR promoted cardiac apoptosis and fibrosis. In hereditary epileptic rats, it was shown that the CaSR protein, cardiac apoptosis, and fibrosis were increased, thus inducing the activation of the MAPK, transforming growth factor beta (TGF-β), and connective tissue growth factor (CTGF) pathways, which were aggravated by GdCl_3_ and attenuated by NPS-2390 [88]. In primary neonatal rat cardiac fibroblasts, high glucose induced myocardial fibrosis via the upregulation of the TGF-β1/Smads pathway, which was promoted by the CaSR agonist R568 but reduced by the CaSR inhibitor Calhex231 [89]. In T1D rats, Calhex231 could inhibit the TGF-β1/Smads pathway and then depress the proliferation of cardiac fibroblasts, along with the reduction deposition of collagen, alleviating glucose-induced myocardial fibrosis [60]. Collectively, these results suggested that TGF-β1 pathways were involved in the CaSR-promoted fibrosis of cardiac fibroblasts, but this needs to be further confirmed by investigating the special inhibitors of the pathways, and their downstream effectors also need to be identified. Engrailed 1 (EN1) re-expressed in multiple fibroblast subpopulations as a molecular amplifier of TGFβ signaling in myofibroblast differentiation, and a coordinator of the reorganization of the cytoskeleton during myofibroblast differentiation [90,91,92]. Thus, the targeted inactivation of EN1 may be a potential strategy to inhibit TGFβ signaling and fibroblast activation in fibrotic diseases.

(e) The hypoxia-associated activation of NFkB leads to IRR. Hypoxia occurred in a number of pathological conditions, such as MI, diabetes, obesity, myocardial ischemia, and cardiac hypertrophy. A hypoxia condition can dramatically increase the cytosolic Ca^2+^ levels and induce necrosis of cells and then release alarmins, which would trigger the activation of NFkB signaling to induce the IRR of the adaptation cells [93]. Chronic intermittent hypoxia-induced cardiac inflammation, apoptosis, and fibrosis in a rat obstructive sleep apnea model were associated with the increased expression of NFkB signaling molecules [94]. In neonatal rat ventricular myocytes and transverse aortic constriction mice, the hypoxia-induced development of cardiac hypertrophy could be prevented by inhibiting the CaSR with Calhex231 [95]. It has been indicated that the CaSR in human peripheral blood T lymphocytes is involved in the AMI onset and progression and promotes IL-6 and TNF-β releases and their mRNA expressions through the NFκB signaling pathway [96,97]. In addition, the CaSR gene transcription was upregulated by IL-1β and TNF-α via NFkB in kidney proximal tubule cells [98]. However, the study of the CaSR regulation of NFkB signaling under hypoxia stress is still limited.

### 3.2. CaSR/Wnt/β-Catenin Signaling

The activation of Wnt/β-catenin signaling is associated with chronic inflammation and organ fibrosis. Wnt/β-catenin signaling was s upregulated in CKD patients and contributed to remodeling and myocardial fibrosis [99,100]. The inhibition of the Wnt/β-catenin pathway ameliorated CKD-associated vascular calcification, which could be reversed by the PPARγ antagonist GW9662 [101]. CaSR activation modulated the crosstalk between Wnt and PPARγ pathways in adipocytes [102]. The CaSR was revealed to be co-distributed with β-catenin in Madin–Darby canine kidney (MDCK) cells and the β-catenin nuclear localization depended on the activation and expression of the CaSR in colonic epithelial cells [103,104]. It was reported that Wnt/β-catenin signaling mediated both heart and kidney inflammation and fibrosis in type 2 CRS [105]. However, the regulation of inflammation and fibrosis by CaSR/Wnt/β-catenin signaling in the hear-kidney interaction still needs to be clarified.

### 3.3. Autophagy–Lysosomal Pathway

The CaSR can modulate cell processes through the autophagy-lysosomal pathway, which has great potential as a treatment for damaged kidneys and cardiomyocyte injury. The CaSR-mediated autophagy pathway was not involved in the I/R-induced cardiomyocyte apoptosis [106]. Interestingly, both in vivo and in vitro studies suggested that the CaSR inhibitor Calhex231 inhibited autophagy by suppressing the CaMKKβ-AMPK-mammalian target of rapamycin (mTOR) pathway to ameliorate isoproterenol or Ang II-induced cardiomyocyte hypertrophy and cardiac fibrosis [107,108,109]. In kidney cells, it was shown that the CaSR stimulated the chaperone Hsp70-assisted protein degradation of inflammasome assemblage, including ASC, NLRP3, IRAK1, and TRAF6, through the combination of the ubiquitin–proteasome pathway, endosomal microautophagy, and macroautophagy [110]. Cardiovascular disorders are the most common causes of mortality in autosomal dominant polycystic kidney disease (ADPKD), which is mainly caused by the deficiency of polycystin-1 (PC1) or polycystin-2 (PC2) [111]. The lack of PKD1 impaired lysosomal acidification in a calpain protease-dependent manner, and this altered autophagy flux contributed to ADPKD progression [112]. By using the conditionally immortalized proximal tubular epithelial cells deficient for polycystin-1 (ciPTEC-PC1KD), it was shown that the calcimimetic NPS R-568-activated CaSR could reduce the mTOR activity and restore the lower mitochondrial calcium levels caused by PC1 deficiency, recover the mitochondrial ATP production and repair oxidative phosphorylation [113]. Our previous studies suggested that the activation of the CaSR by calcimimetic compound NPS R-467 could protect against the heavy metal cadmium-induced mouse kidney injury and cell apoptosis by restoring the autophagy–lysosome process [5,23].

### 3.4. Potential Target of Exosomes in Cardiorenal Syndrome

Due to their role in ischemic signaling, myocardial repair, and communication between the heart and the bone marrow, exosomes are considered novel targets in the CRS puzzle and the crosstalk between the heart and the kidneys [71,72]. The bone marrow mesenchymal stem cell-derived exosomes protected against myocardial infarction by promoting autophagy and inhibiting cell proliferation and migration, as well as cell apoptosis during hypoxia–reoxygenation [114]. However, the mechanism of the autophagy-related proteins involved was not identified. Although the production and function of exosomes in kidney tissues and cells remain unclear, the production of exosomes during hypoxia is protective in renal tubular cells [115]. In vitro, TGF-β1 stimulated kidney proximal tubular cells to produce exosome, which could induce renal interstitial fibroblast activation but was abolished by the exosome secretion inhibitor dimethyl amiloride. In vivo, the injections of tubular-cell-derived exosomes aggravated kidney injury and fibrosis, while these conditions were ameliorated by blocking exosome secretion [116]. In addition, the exosomes from endothelial progenitor cells improved survival, by suppressing renal vascular leakage and reducing kidney dysfunction in septic mice [117]. However, the mechanisms and methodologies of exosomal research in the context of cardiorenal pathology are still limited.

As a Ca^2+^ release channel on lysosomes, the transient receptor potential mucolipin 1 (TRPML1) channel plays an important role in maintaining cardiovascular and renal glomerular homeostasis and thereby contributes to some cardiovascular and kidney diseases. In arterial myocytes, TRPML1 induced intracellular Ca^2+^ homeostasis through an SR Ca^2+^ release. In podocytes, the TRPML1 channel controls lysosome trafficking and exosome release, and its deficiency contributes to podocyte dysfunction [118]. However, the role of CaSR-mediated intracellular Ca^2+^ in the exosome function is still unclear. An in vivo study suggested that the exosomes from CaSR-stimulated polymorphonuclear cells (PMNs) protected against AMI and reperfusion injury in the myocardial tissue by decreasing apoptosis, ROS production, and hypoxia-reoxygenation [30]. It was also shown that this might be related to the AKT signaling pathway, but the potential role of the CaSR–AKT signaling pathway in regulating autophagy needs to be further clarified.

## 4. Conclusions

**Figure 2:**
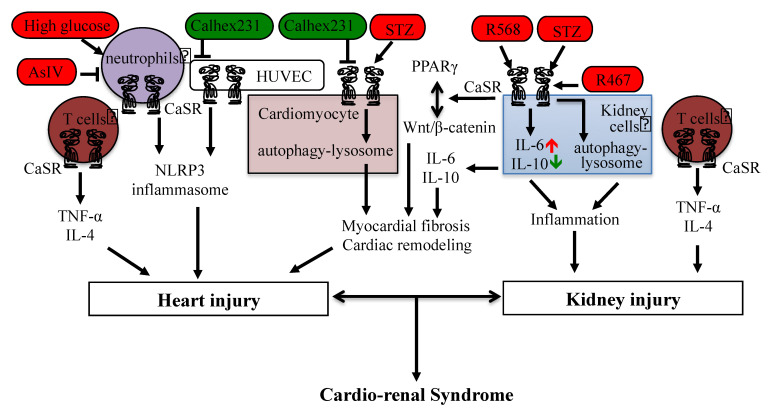
CaSR-mediated signaling in cardiorenal syndrome and heart–kidney interaction. The activation regulator is in red color, and the negative modulator is in green color.

The existing studies demonstrate that the CaSR mediates intracellular communications between the ER, mitochondria, nucleus, protease/proteasome, and autophagy–lysosome, and therefore, it plays complex roles in cardiovascular diseases, including the ischemia/reperfusion-induced myocardial injury, hypertension/hypoxia-induced cardiac hypertrophy, DCM, and cardiorenal syndrome. Moreover, it was also demonstrated that multiple regulations of the CaSR, such as upregulation/downregulation, activation/inhibition, mutation, degradation, or limited proteolytic cleavages, promote or prevent related cardiovascular diseases. In addition, the CaSR-mediated intracellular signaling or pathways contribute to inflammation and fibrosis and are involved in the heart–kidney interaction (Figure 2), which certainly does not rule out the interactions of the heart with other tissues.

As the CaSR plays diverse and crucial roles in human physiology and pathophysiology, both in calcitropic and non-calcitropic tissues and cells, the drugs targeting the CaSR to treat human diseases, including cardiovascular diseases, have been considered. Various positive and negative allosteric modulators of the CaSR have been developed, which can increase or inhibit the sensitivity of this receptor by changing the conformation of the receptor. As cited in this review, the pharmaceutical activation or inhibition of the CaSR might be potential therapeutic strategies to prevent or treat vascular or cardiac diseases. However, both the heart and the kidneys are composed of various cells that have multiple crosstalk pathways and may perform diverse responses to these treatments. Even when considering the approved drugs, such as cinacalcet and etelcalcetide, which are applied for treating secondary hyperparathyroidism in cases of CKD and hypercalcemia with parathyroid carcinoma, it should be noted that etelcalcetide has some adverse reactions that seriously worsen heart failure and cardiovascular system. With the benefits derived from the studies of the CaSR structure, researchers can design more special drugs targeting the CaSR, and more advanced experimental tools and models will be helpful for screening the efficacy of therapeutic strategies [119,120]. We believe that in-depth research on the special CaSR signaling pathways in cellular compartments and targeting these pathways may lead to novel therapeutic strategies against related disorders or diseases.
